# Core skin DC signatures control immune tolerance to skin cancer and limit anti-tumor immunity

**DOI:** 10.1186/2051-1426-3-S2-P205

**Published:** 2015-11-04

**Authors:** Christopher Nirschl, Yong Liu, Kavita Sarin, Mayte Suarez-Farinas, David Chau, Peter Sage, Arlene Sharpe, Niroshana Anandasabapathy

**Affiliations:** 1 Brigham and Women's Hospital, Department of Dermatology, Harvard Medical School, Boston, MA, USA; 2 Stanford University, Stanford, CA, USA; 3 Mount Sinai school of Medicine, New York, NY, USA; 4 Harvard Medical School, Boston, MA, USA

## Background

Dendritic cells (DC) are gatekeepers of immunity, critical to both an initiate immune response upon infection and promote tolerance to self-antigens.

## Methods

We have just established that DC subsets in the skin that constitutively migrate into LNs can individually and collectively temper an on-going immune response. Tissue DC originating in skin share a unique transcriptome signature geared towards immune dampening when compared to their lymphoid counterparts in both mouse and human.

## Results

Here we demonstrate expression of unique core skin DC transcripts are closely associated with increased clinical aggressiveness of BCC in humans and the stratify stage 4 melanoma outcomes. In mice, loss of signature genes in DC, which include but are not limited to PD-L1/PD-L2, lead to enhanced antigen-specific immunity, decreased tumor growth, and improved anti-tumor vaccine priming to melanoma skin cancer by distinguishable molecular control of T cell effector function and clonal proliferation.

## Conclusions

These data suggest core skin DC signatures regulate the immune-epithelial interface.

